# Effects of berbamine against myocardial ischemia/reperfusion injury: Activation of the 5' adenosine monophosphate‐activated protein kinase/nuclear factor erythroid 2‐related factor pathway and changes in the mitochondrial state

**DOI:** 10.1002/biof.1820

**Published:** 2022-02-07

**Authors:** Chennian Xu, Yang Liu, Jian Yang, Mengen Zhai, Zhenge Fan, Rui Qiao, Ping Jin, Lifang Yang

**Affiliations:** ^1^ Department of Cardiovascular Surgery General Hospital of Northern Theater Command Shenyang China; ^2^ Department of Cardiovascular Surgery Xijing Hospital, Air Force Medical University Xi'an China; ^3^ Department of Anesthesiology Xi'an Children's Hospital Xi'an China

**Keywords:** AMPK/Nrf2, berbamine, heart, ischemia/reperfusion injury, mitochondrion, oxidative stress

## Abstract

This study was designed to investigate whether berbamine (BA)‐induced cardioprotective effects were related to 5′ adenosine monophosphate‐activated protein kinase (AMPK)/nuclear factor erythroid 2‐related factor (Nrf2) signaling and changes in the mitochondria in myocardial ischemia/reperfusion (I/R) injury. C57/BL6 mice were exposed to BA (10 mg/kg/d), with or without administration of the AMPK specific inhibitor compound C (5 mg/kg/d) or the Nrf2 specific inhibitor ML‐385 (30 mg/kg/d), and then subjected to a myocardial I/R operation. As expected, BA significantly improved post‐ischemic cardiac function, reduced infarct size and apoptotic cell death, decreased oxidative stress, and improved the mitochondrial state. Furthermore, BA markedly increased AMPK activation, Nrf2 nuclear translocation, and the levels of NAD(P)H quinone dehydrogenase and heme oxygenase‐1. Nevertheless, these BA‐induced changes were abrogated by compound C. In addition, ML‐385 also canceled the cardioprotective effects of BA but had little effect on AMPK activation. Our results demonstrate that BA alleviates myocardial I/R injury and the mitochondrial state by inhibiting apoptosis and oxidative stress via the AMPK/Nrf2 signaling pathway.

**Abbreviations**
ACC: acetyl‐CoA carboxylase
AMPK: 5′ adenosine monophosphate‐activated protein kinase
BA: berbamine
CK: creatine kinase
ELISA: enzyme‐linked immunosorbent assay
GAPDH: glyceraldehyde 3‐phosphate dehydrogenase
HO‐1: heme oxygenase‐1
I/R: ischemia/reperfusion
LDH: lactate dehydrogenase
LVEF: left ventricular ejection fraction
LVFS: left ventricular fraction shortening
NQO‐1: NAD(P)H quinone dehydrogenase
Nrf2: nuclear factor erythroid 2‐related factor
ROS: reactive oxygen species
SEM: standard error of the mean
SOD: superoxide dismutase
TUNEL: terminal deoxynucleotidyl transferase‐mediated dUTP nick end labeling

## 1 INTRODUCTION

Ischemic heart disease is the leading cause of morbidity and mortality in the United States and other parts of the world.^1^ For patients presenting with ST‐segment elevation myocardial infarction, timely reperfusion, using primary percutaneous coronary intervention or thrombolytic therapy, is the treatment of choice for preserving left ventricular function and limiting myocardial infarct size.^2^, ^3^ However, the process of restoring coronary blood flow to the ischemic cardiac tissue can, in itself, induce myocardial injury and cardiomyocyte death through a burst of reactive oxygen species (ROS) production, inflammatory response, calcium overload, and mitochondrial dysfunction, a phenomenon termed ischemia/reperfusion (I/R) injury.^4^, ^5^ Oxidative stress is considered to be the key pathogenic mechanism of myocardial I/R injury.^6^, ^7^ Disruption of oxidant‐antioxidant homeostasis, such as ROS generation and the inhibition of superoxide dismutase (SOD) activity, is the main factor responsible for cellular oxidative stress. Therefore, illustrating the molecular mechanisms that could inhibit oxidative stress may be useful to prevent myocardial I/R injury. At present, researchers have found that mitochondrial structural damage and function reduction are the main mechanisms of myocardial I/R injury.^8^ More and more attention has been paid to the role of mitochondrial regulation and protection in myocardial I/R injury.^9^ There are still many problems to be solved about the protective effect of mitochondrial injury and oxidative stress in the process of myocardial I/R injury. For example, appropriate mitochondrial autophagy can remove damaged mitochondria in time to avoid the release of oxygen free radicals from damaged mitochondria and aggravate cell injury.^10^ PINK1 can rapidly accumulate on the outer membrane surface of damaged mitochondria. On one hand, it can promote damaged mitochondria to be swallowed by autophagosomes by recruiting autophagy receptors such as p62 and binding with LC3B to form autophagosomes. On the other hand, mitochondrial autophagy can be induced by directly activating parkin and mediating mitochondrial ubiquitin chain synthesis.^11^ Studies have shown that after myocardial I/R and the recovery of the oxygen supply, calcium overload and an ROS surge change the permeability of the mitochondrial membrane, leading to the activation of cytochrome and protein kinase, mitochondrial swelling, and apoptosis, thus finally leading to apoptosis and loss of function.^12^, ^13^

5′Adenosine monophosphate‐activated protein kinase (AMPK), a serine/threonine kinase, has been identified as a pivotal energy sensor and regulator of cellular metabolism that has an important role in the regulation of energy homeostasis and of the reduction in ROS production under normal and ischemic conditions.^14^, ^15^ Activation of AMPK occurs during cellular energetic stress, including ischemia/hypoxia, due to the changes in the intracellular AMP/ATP ratio.^16^ As a consequence, AMPK activation ameliorates the pathogenesis of metabolic disorders by regulating the expression and activation of various downstream molecules.^17^ In addition to regulating metabolism, AMPK also participates in the regulation of many other cellular processes, including autophagy, apoptosis, endoplasmic reticulum stress, inflammatory responses, and oxidative stress.^18^, ^19^, ^20^ Nuclear factor erythroid 2‐related factor (Nrf2) is an important transcription factor that translocates into the nucleus and controls the expression of many target genes.^21^ It is well known that Nrf2 plays an essential role in balancing the oxidants and antioxidants.

Berbamine (BA) is a natural compound of Berberis and its derivatives, which have been proved to have an antitumor effect. BA‐induced apoptosis exists mainly in a wide range of cancer cells and tumors. The potential mechanism of BA‐induced cell apoptosis and the activity of cancer cells may be related to the downregulation of bcr/abl gene expression and change in the mitochondrial membrane. BA derivatives can improve cell proliferation of ROS and target calcium/calmodulin‐dependent death protein kinase II. Therefore, BA may play a potential protective role in antitumor and cardiovascular protection.^22^, ^23^, ^24^, ^25^ Previous studies have shown that BA prevents cardiomyocyte death and injury by inhibiting mitochondrial oxidative stress and the inflammatory response.^26^ Additionally, Sharma et al. indicated that the protective effects of the preoperative application of BA are related to the AMPK activation/regulation of the mTOR/SREBP‐1c axis and the Nrf2/ antioxidant response element pathway to allay lipid accumulation and oxidative stress in steatotic HepG2 cells.^27^ However, the mechanisms underlying the regulation of Nrf2 by BA have not been clarified. Therefore, the goal of this study was to determine the protective actions of BA against myocardial I/R injury and determine whether AMPK/Nrf2 signaling is associated with BA‐induced cardioprotective effects and mitochondrial regulation and protection.

## 2 MATERIALS AND METHODS

### 2.1 Animals

Male C57/BL6 mice (8 weeks old, 20–25 g body weight) were provided by the Laboratory Animal Center at the Air Force Medical University and housed in a controlled environment (22–25°C; 55%–60% humidity; 12‐h light/dark cycle; free access to food and water). All procedures were performed in accordance with the Guidelines for the Care and Use of Laboratory Animals of the National Academy of Sciences and published by the National Institutes of Health (NIH Publication No. 80‐23, revised in 1996). All experimental protocols were reviewed and approved by the Research Commission on Ethics of the Air Force Medical University.

### 2.2 Chemicals and reagents

Berbamine, Evans blue, triphenyltetrazolium chloride, and 4′,6‐diamidino‐2‐phenylindole dihydrochloride were purchased from Sigma‐. Enzyme‐linked immunosorbent assay (ELISA) kits for detecting lactate dehydrogenase (LDH), SOD, and creatine kinase (CK) activities and malondialdehyde (MDA) content were purchased from the Institute of Nanjing Jiancheng Bioengineering Institute. The terminal deoxynucleotidyl transferase‐mediated dUTP nick end labeling (TUNEL) assay kit was purchased from Roche Biochemicals. Primary antibodies against AMPK, phospho‐AMPK, acetyl‐CoA carboxylase (ACC), phospho‐ACC, Nrf2, heme oxygenase‐1 (HO‐1), and NAD(P)H quinone dehydrogenase (NQO‐1) were purchased from Abcam. A primary antibody against SOD2 (sc‐30080) was obtained from Santa Cruz Biotechnology. Primary antibodies against cytochrome c, cleaved caspase‐3, gp91^phox^, histone H3, and glyceraldehyde 3‐phosphate dehydrogenase (GAPDH) were purchased from Cell Signaling Technology. Goat anti‐rabbit and goat anti‐mouse secondary antibodies were purchased from the Zhongshan Company. Dihydroethidium, which detects ROS generation in cardiac tissues, was purchased from Invitrogen. Compound C and ML385 were purchased from Selleckchem.

### 2.3 Myocardial ischemia/reperfusion protocol

The myocardial I/R injury model was used as described previously.^26^ Briefly, mice were anesthetized using pentobarbital sodium (50 mg/kg) by intraperitoneal injection and ventilated via tracheal intubation with a Harvard rodent respirator (Harvard Apparatus). Body temperature was maintained at 37°C via a heated operating table. The left anterior descending coronary artery was ligated by placing a 6–0 silk suture and making a slip knot. Myocardial I/R injury was carried out by inducing ischemia for 30 min following by reperfusion for 2 h (for TUNEL and western blot assay) or 24 h (for echocardiographic, hemodynamic, and infarct size measurements). The sham group underwent the same operative procedures but the left anterior descending coronary artery was not tied. The mice were randomly divided into the following groups: (1) sham group; (2) I/R group; (3) BA+I/R group; (4) BA+compound C + I/R group; and (5) BA+ML‐385 + I/R group. BA (20 mg/kg) was injected intraperitoneally 10 min prior to reperfusion. Compound C (0.25 mg/kg) and ML‐385 (30 mg/kg) were injected intraperitoneally 15 min prior to reperfusion. The concentrations of BA, compound C, and ML‐385 used were based on information from previous studies.^26^, ^28^, ^29^, ^30^ The mice in the sham group were injected intraperitoneally daily with saline at the same volume.

### 2.4 Echocardiographic measurements

As described in a previous study,^31^ heart function was measured by transthoracic echocardiography with a VisualSonics Vevo 770 echocardiography machine. Briefly, mice were anesthetized with 1% isoflurane and placed on a thermostatic pad. The left ventricular ejection fraction (LVEF) and left ventricular fractional shortening (LVFS) were measured at the level of the papillary muscles by motion‐mode echocardiography. All measurements were based on six consecutive cardiac cycles.

### 2.5 Measurements of myocardial infarct sizes

As described previously,^26^ at the end of reperfusion, the left anterior descending coronary artery was reoccluded, and 2% Evans blue dye was injected into the left ventricular cavity. Then the hearts were quickly frozen at −80°C for 1 h and sectioned horizontally into six slices (2 mm thick) from the apex to the base axis. The slices were incubated in 1% triphenyltetrazolium at 37°C for 15 min in the dark and subsequently fixed in 4% paraformaldehyde overnight at room temperature. The irreversibly injured tissue was stained white; areas at risk were brick red. Myocardial infarct size was determined as the percentage of areas of risk using Image‐Pro Plus software (Media Cybernetics).

### 2.6 Reactive oxygen species measurement

Measurement of ROS was performed as described previously.^31^ The oxidative fluorescent dye dihydroethidium was used to measure intracellular ROS production in the myocardial frozen sections. Fresh‐frozen myocardium was incubated with oxidative fluorescent dye and examined with an Olympus FV1000 (Olympus) laser confocal microscope according to the manufacturer's instruction. The intensity of the fluorescence was calculated using Image‐Pro Plus software (Media Cybernetics).

### 2.7 Determination of the myocardial apoptotic index

Myocardial apoptosis was analyzed by TUNEL staining using an in situ cell death detection kit as described previously.^26^ Apoptotic cell nuclei were stained with the TUNEL reaction mixture, and all nuclei were labeled with 4′,6‐diamidino‐2‐phenylindole dihydrochloride. Images were obtained using an Olympus FV1000 (Olympus). TUNEL‐positive cells showed green fluorescence, and all nuclei displayed blue fluorescence. The percentage of the apoptotic index was calculated as the ratio of the TUNEL‐positive cell nuclei to the total number of nuclei.

### 2.8 Determination of lactate dehydrogenase, creatine kinase, and superoxide dismutase activity, and assessment of malondialdehyde content

The activities of serum LDH and CK were used to estimate myocardial necrosis using ELISA kits, following the manufacturer's instructions. The activity of SOD and the content of MDA in cardiac tissues were determined using ELISA kits from the Institute of Nanjing Jiancheng Bioengineering Institute.

### 2.9 Western blot analysis

Left ventricular tissues were snap‐frozen immediately and stored at −80°C until analyzed. To determine the protein of cytochrome c, a cytosolic fraction was prepared with lysis buffer containing 50 mM Tris‐HCl (pH 7.3), 150 mM NaCl, 5 mM EDTA, 1 mM dithiothreitol, 1% protease inhibitor cocktail, and 1% phosphatase inhibitor cocktail. For others, additional 1% triton X‐100 was added to the lysis buffer to extract the total proteins. After homogenization, the samples were centrifuged at 12,000*g* for 20 min, and the supernatants were collected. Protein concentrations were determined using the bicinchoninic assay. Denatured samples were subjected to either 8% or 12% sodium dodecyl sulfate polyacrylamide gels and transferred onto polyvinylidene fluoride membranes. The membranes were incubated with primary antibodies against phophso‐AMPK (1:1000), AMPK (1:1000), phophso‐ACC (1:1000), ACC (1:1000), Nrf2 (1:1000), NQO‐1 (1:1000), HO‐1 (1:1000), gp91^phox^ (1:1000), cytochrome c (1:1000), cleaved caspase‐3 (1:1000), histone H3 (1:1000), and GAPDH (1:5000), followed by horseradish peroxidase‐conjugated secondary antibody for 1 h at room temperature. After that, the membranes were exposed to the chemiluminescence reaction and imaged with ChemiDoc XRS (Bio‐Rad). Equal protein loading was confirmed by GAPDH probing of membranes. Relative densitometry was analyzed using the Image Lab (Bio‐Rad Laboratories), as described previously.^31^

### 2.10 Transmission electron microscope observation

According to the requirements of electron microscope specimen preparation and sampling, for each specimen, a 1‐mm^3^ section of left ventricular myocardial tissue was cut, quickly put into 2.5% glutaraldehyde solution, and fixed overnight at 4°C. The fixed tissue was sent to the electron microscope room to make specimens. The changes in the mitochondria in the myocardial tissue from each group were observed using a transmission electron microscope and photographed for preservation.

### 2.11 Statistical analysis

All data were expressed as mean ± standard error of the mean. Statistical analyses were performed using GraphPad Prism (GraphPad Software, Inc.). The statistical significance of the differences was determined by a Student *t* test between two groups or one‐way analysis of variance followed by Bonferroni's multiple comparison for a post hoc *t*‐test. A value of *p* < 0.05 was considered to be statistically significant.

## 3 RESULTS

### 3.1 Compound C abolished berbamine‐induced alleviation of infarct size and cardiac function during myocardial ischemia/reperfusion

**FIGURE 1:**
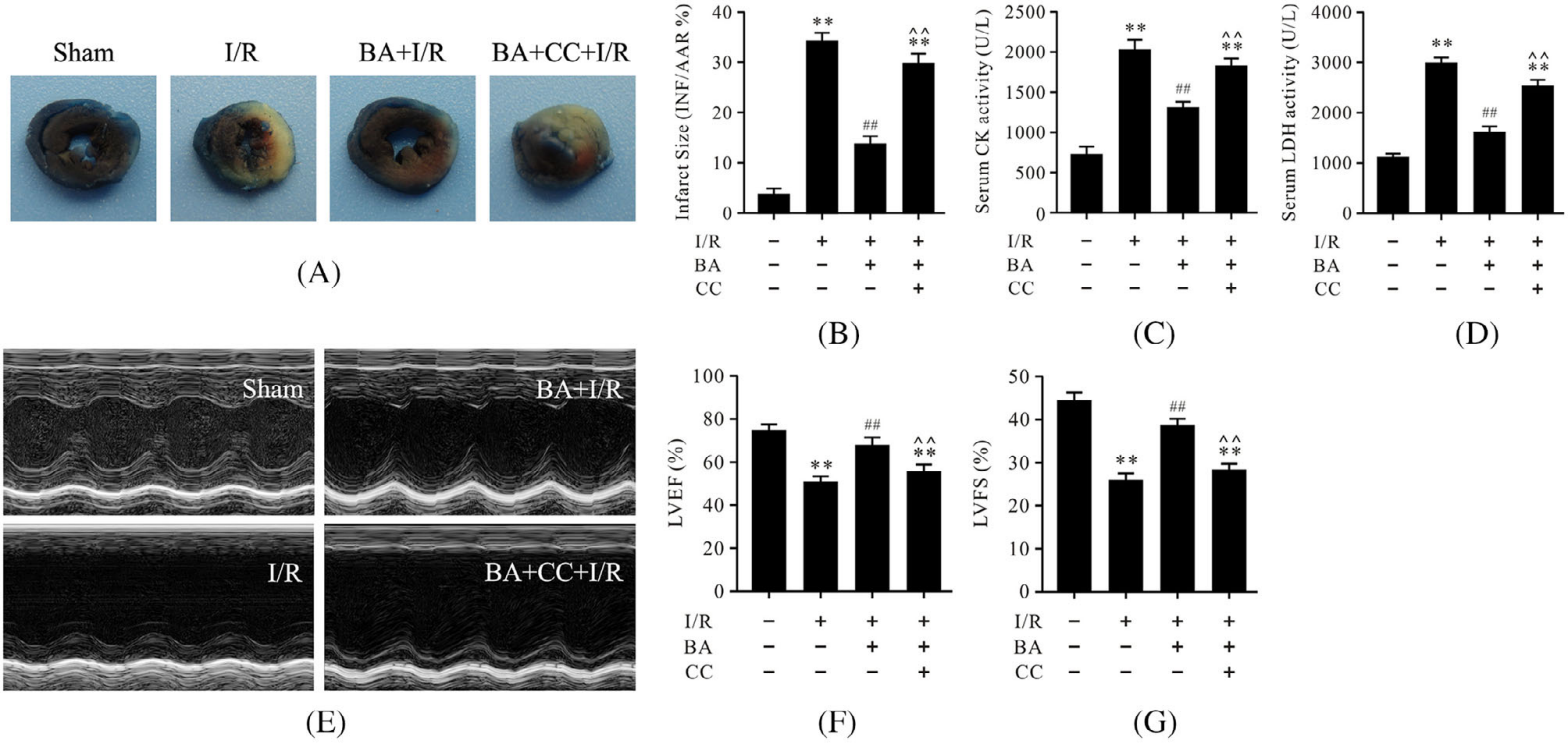
Compound C abrogated the cardioprotective effects of berbamine in myocardial ischemia/reperfusion. (A) Evans blue and TTC staining of heart slices; (B) myocardial infarct size; (C) serum CK activity; (D) serum LDH activity; (E) representative images of motion‐mode echocardiography; (F) LVEF; (G) LVFS. Data are presented as the mean ± SEM, *n* = 6. **p < 0.01 versus the sham group; ^##^ *p* < 0.01 versus the I/R group; **^^** *p* < 0.01 versus the BA+I/R group. BA, berbamine; CC, compound C; CK, creatine kinase; I/R, ischemia/reperfusion; LDH, lactate dehydrogenase; LVEF, left ventricular ejection fraction; LVFS, left ventricular fraction shortening; SEM, standard error of the mean. TTC, triphenyltetrazolium chloride

To explore the role of BA in myocardial I/R and to determine whether the AMPK pathway is involved in the cardioprotective effects of BA, compound C, the AMPK specific inhibitor, was used in this experiment, and cardiac function and myocardial injury were subsequently evaluated. As revealed in Figure 1A,B, I/R treatment dramatically increased infarct size compared to the sham group. Moreover, the serum CK and LDH activities were also significantly increased in the I/R group (Figure 1C,D). Meanwhile, as shown in Figures 1E–G, the results of echocardiography showed that LVEF and LVFS, two major indicators of cardiac function, were markedly decreased in the I/R group. As expected, BA treatment significantly improved cardiac function and reduced myocardial injury (Figure 1A–G). In particular, compound C significantly obstructed the cardioprotective effects of BA in myocardial I/R (Figure 1A–G).

### 3.2 Compound C prevented berbamine from exerting antiapoptotic effects on cardiomyocytes during myocardial I/R

**FIGURE 2:**
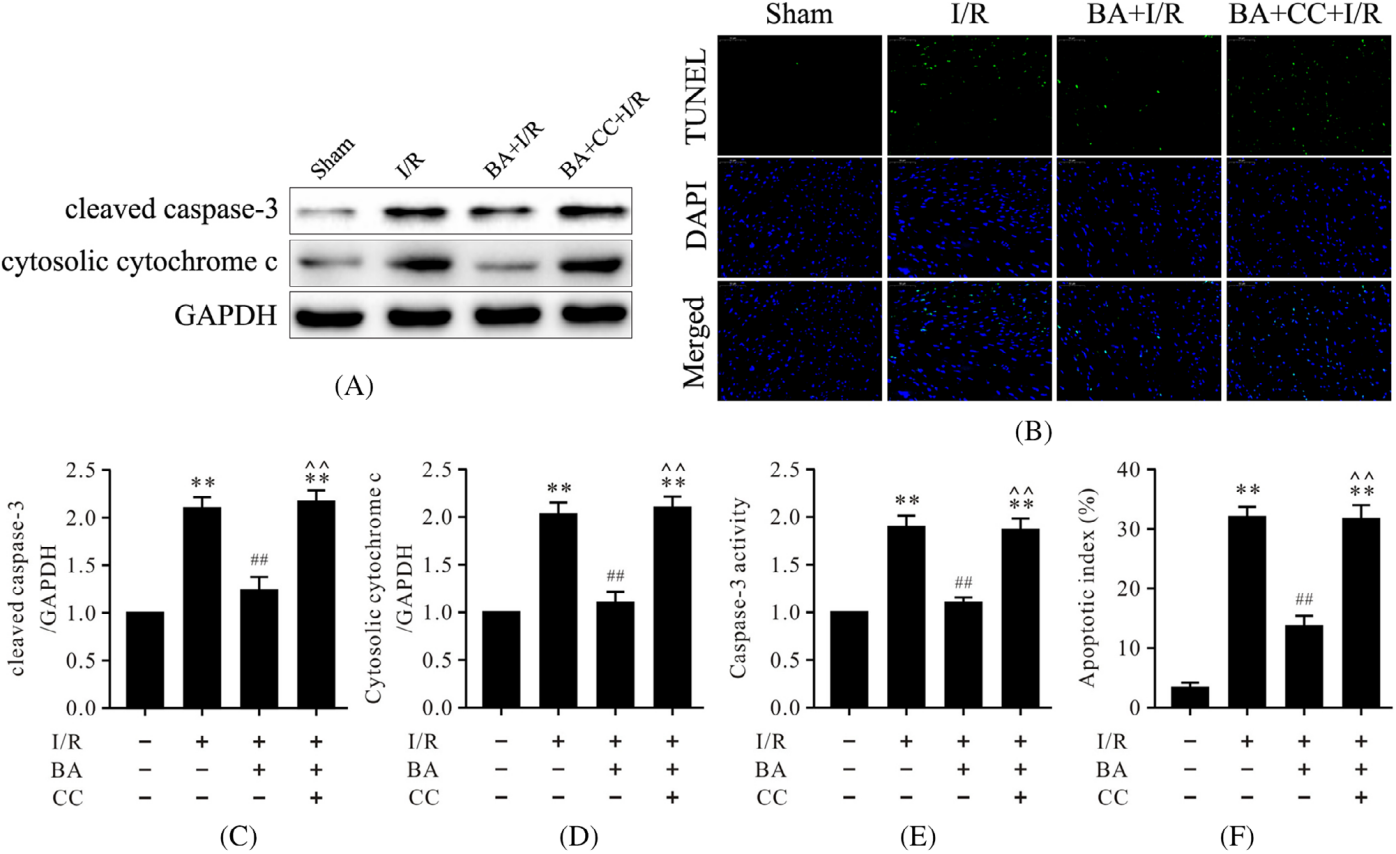
Compound C abolished the antiapoptotic effects of BA in myocardial I/R injury. (A) Representative immunoblots of cleaved caspase‐3, cytosolic cytochrome c, and GAPDH (internal control); (B) representative images of TUNEL staining of left ventricular tissue; (C,D) semiquantitative analysis of cleaved caspase‐3 and cytosolic cytochrome c; (E) caspase‐3 activity; (F) quantitative analysis of apoptotic index (magnification, **×**400). Data are presented as the mean ± SEM, *n* = 6. **p < 0.01 versus the sham group, ^##^ *p* < 0.01 versus the I/R group, **^^** *p* < 0.01 versus the BA+I/R group. BA, berbamine; CC, compound C; GAPDH, glyceraldehyde 3‐phosphate dehydrogenase; I/R, ischemia/reperfusion; SEM, standard error of the mean; TUNEL, terminal deoxynucleotidyl transferase‐mediated dUTP nick end labeling

It is well known that apoptosis is the main characteristic of myocardial I/R, which prompted us to assess the antiapoptotic effects of BA on cardiomyocytes during I/R. As illustrated in Figure 2, western blot analysis showed that the expressions of cleaved caspase‐3 and cytosolic cytochrome c were notably increased in the I/R group compared with the sham group Figure 2A,C,D). The apoptotic index and caspase‐3 activity were also up‐regulated in the I/R group (Figure 2B,E,F). Treatment with BA could inhibit myocardial apoptosis following I/R, as evidenced by the notably decreased expressions of cleaved caspase‐3 and cytosolic cytochrome c and the significantly reduced apoptotic index and caspase‐3 activity (Figure 2A–F). It was important to note that the antiapoptotic effects of BA during myocardial I/R were attenuated by compound C (Figure 2A–F).

### 3.3 Compound C inhibited antioxidative effects of berbamine during myocardial ischemia/reperfusion

**FIGURE 3:**
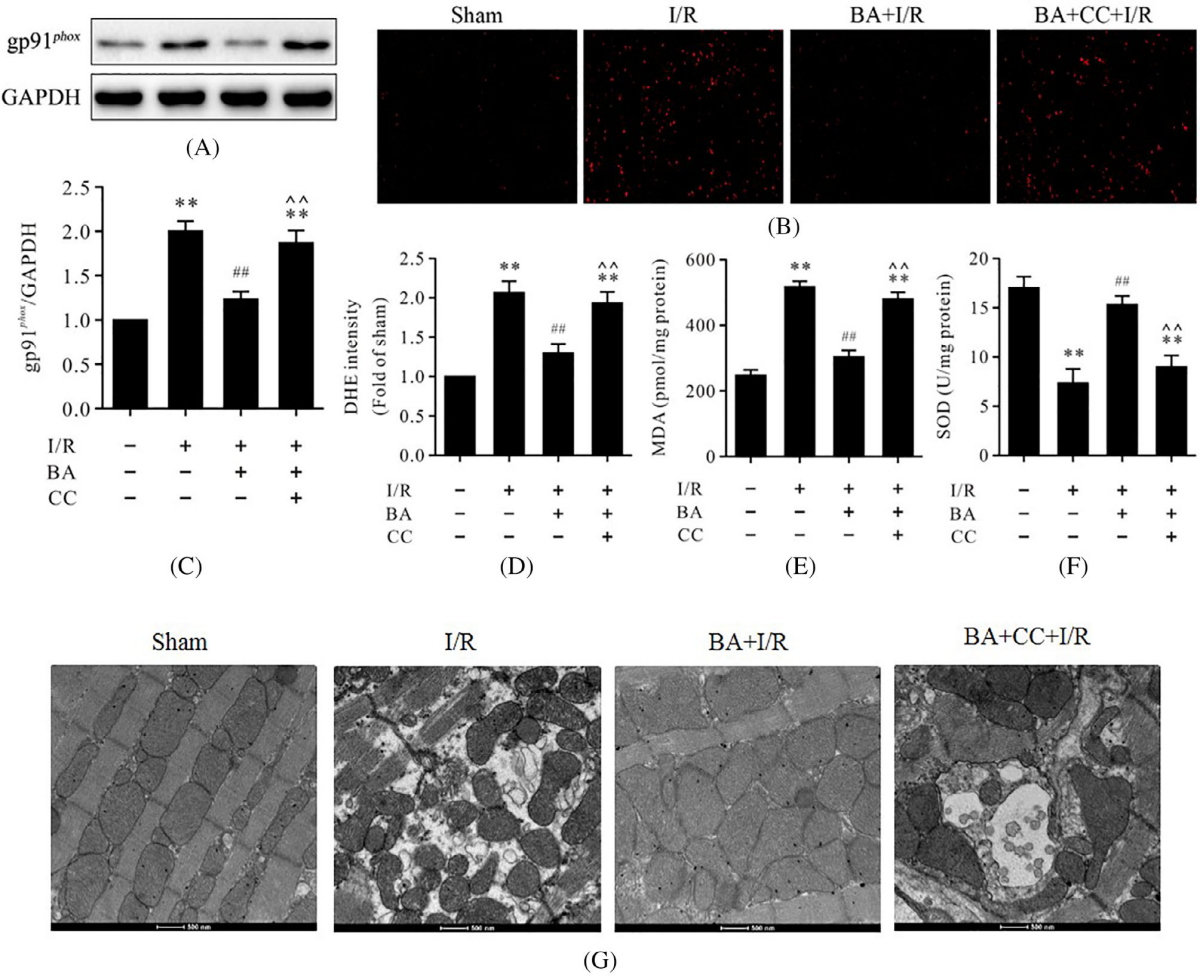
Compound C obstructed the antioxidative effects of BA and the mitochondrial state in myocardial I/R injury. (A) Representative immunoblots of gp91^phox^ and GAPDH (internal control); (B) representative images of DHE staining of left ventricular tissue (magnification, ×400); (C) semi‐quantitative analysis of gp91^phox^; (D) DHE intensity; MDA contents; (F) SOD activity in myocardial tissues; and (G) mitochondrial morphology and state. Data are presented as the mean ± standard error of the mean, *n* = 6. **p < 0.01 versus the sham group, ^##^ *p* < 0.01 versus the I/R group, **^^** *p* < 0.01 versus the BA+I/R group. BA, berbamine; CC, compound C; DHE, dihydroethidium; GAPDH, glyceraldehyde 3‐phosphate dehydrogenase; I/R, ischemia/reperfusion; MDA, malondialdehyde

Oxidative stress has been demonstrated to play a vital role in myocardial I/R injury. To explore the effects of BA on oxidative stress in myocardial I/R, the levels of gp91^phox^, ROS, MDA, and SOD activity were detected. As shown in Figure 3A,C, western blot analysis showed that the protein level of gp91^phox^, an important oxidative stress marker, was remarkably increased in the I/R group compared to the sham group. The levels of MDA and ROS were also significantly increased in the I/R group whereas the SOD activity was decreased (Figure 3B,D–F), which suggested that oxidative stress was grievous in the I/R group. Interestingly, treatment with BA could significantly lower the levels of MDA and ROS and increase SOD activity compared to the I/R group (Figure 3A–F), suggesting that BA alleviated oxidative stress in myocardial I/R. However, compound C could blunt the antioxidative effects of BA during myocardial I/R (Figure 3A–F). Transmission microscope observation can directly reflect the changes in the mitochondria. The electron microscope results showed that the morphology and structure of mitochondria in the sham group were normal and that the density of the matrix was uniform and arranged neatly. In the I/R group, the mitochondria were swollen and deformed, the cristae were broken and had disappeared, and the arrangement was disordered. Compared with the I/R group, the morphology of mitochondria in the BA + I/R group recovered, the mitochondrial cristae were roughly normal and slightly swollen, and the structure was improved whereas compound C could reverse these effects induced by BA (Figure 3G).

### 3.4 Compound C attenuated berbamine‐induced AMPK activation and Nrf2 nuclear translocation in myocardial ischemia/reperfusion injury

**FIGURE 4:**
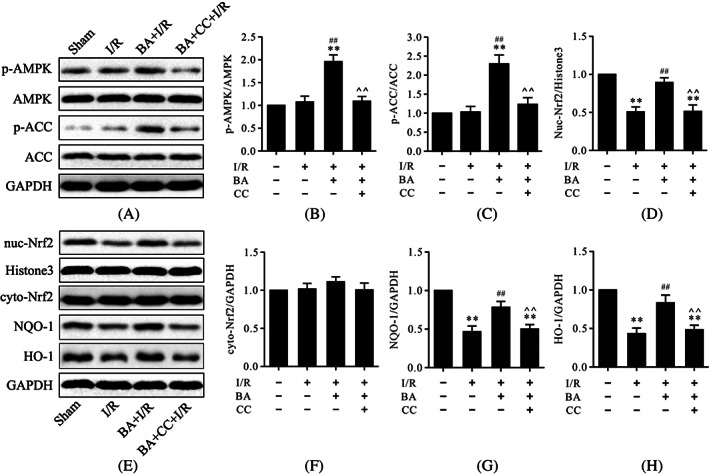
Compound C prevented BA‐induced AMPK activation and Nrf2 nuclear translocation in myocardial I/R injury. (A) Representative immunoblots of p‐AMPK, AMPK, p‐ACC, ACC, and GAPDH (internal control); (B,C) semiquantitative analysis of p‐AMPK and p‐ACC; (D) semiquantitative analysis of nuc‐Nrf2; (E) representative immunoblots of nuc‐Nrf2, histone H3 (internal control), cyto‐Nrf2, NQO‐1, HO‐1, and GAPDH (internal control); (F–H) semiquantitative analysis of cyto‐Nrf2, NQO‐1, and HO‐1. Data are presented as the mean ± standard error of the mean, *n* = 6. **p < 0.01 versus the sham group, ^##^ *p* < 0.01 versus the I/R group, **^^** *p* < 0.01 versus the BA+I/R group. BA, berbamine; CC, compound C; cyto‐Nrf2, cytoplasmic Nrf2; GAPDH, glyceraldehyde 3‐phosphate dehydrogenase; I/R, ischemia/reperfusion; nuc‐Nrf2, intranuclear Nrf2

The AMPK/Nrf2 pathway is described as an important mechanism in protecting against oxidative stress‐induced injury. To investigate the molecular mechanism underlying the antioxidative effects of BA, western blot was used to evaluate the protein levels of p‐AMPK, p‐ACC, NQO‐1, HO‐1, cytoplasmic Nrf2, and intranuclear Nrf2. As shown in Figure 4, western blot analysis showed that I/R treatment could significantly reduce the protein levels of NQO‐1, HO‐1, and intranuclear Nrf2 but had little effect on the protein levels of p‐AMPK, p‐ACC, and cytoplasmic Nrf2 compared to the sham group (Figure 4). More importantly, treatment with BA could significantly increase the protein levels of p‐AMPK, p‐ACC, NQO‐1, HO‐1, and intranuclear Nrf2, which suggested that BA strengthened the activation of the AMPK/Nrf2 pathway in myocardial I/R (Figure 4). However, compound C could reverse these effects induced by BA (Figure 4).

### 3.5 ML‐385 inhibited antiapoptotic and antioxidative effects of berbamine during myocardial ischemia/reperfusion

**FIGURE 5:**
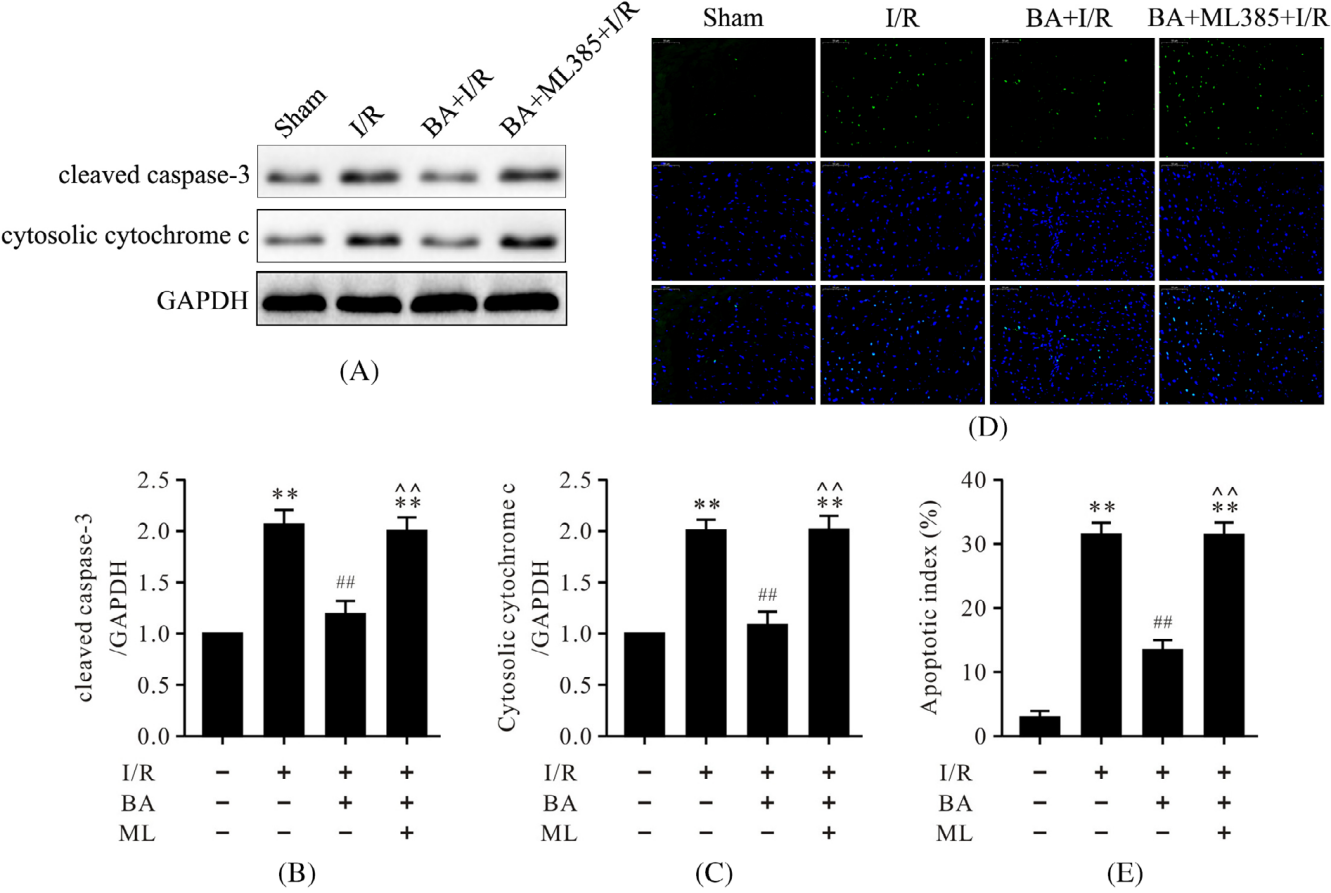
ML‐385 abolished the antiapoptotic effects of BA in myocardial I/R injury. (A) Representative immunoblots of cleaved caspase‐3, cytosolic cytochrome c, and GAPDH (internal control); (B,C) semiquantitative analysis of cleaved caspase‐3 and cytosolic cytochrome c; (D) representative images of TUNEL staining of left ventricular tissue; (E) quantitative analysis of apoptotic index (magnification, ×400). Data are presented as the mean ± standard error of the mean, *n* = 6. **p < 0.01 versus the sham group, ^##^ *p* < 0.01 versus the I/R group, **^^** *p* < 0.01 versus the BA+I/R group. BA, berbamine; I/R, ischemia/reperfusion; ML, ML‐385; GAPDH, glyceraldehyde 3‐phosphate dehydrogenase; TUNEL, terminal deoxynucleotidyl transferase‐mediated dUTP nick end labeling

**FIGURE 6:**
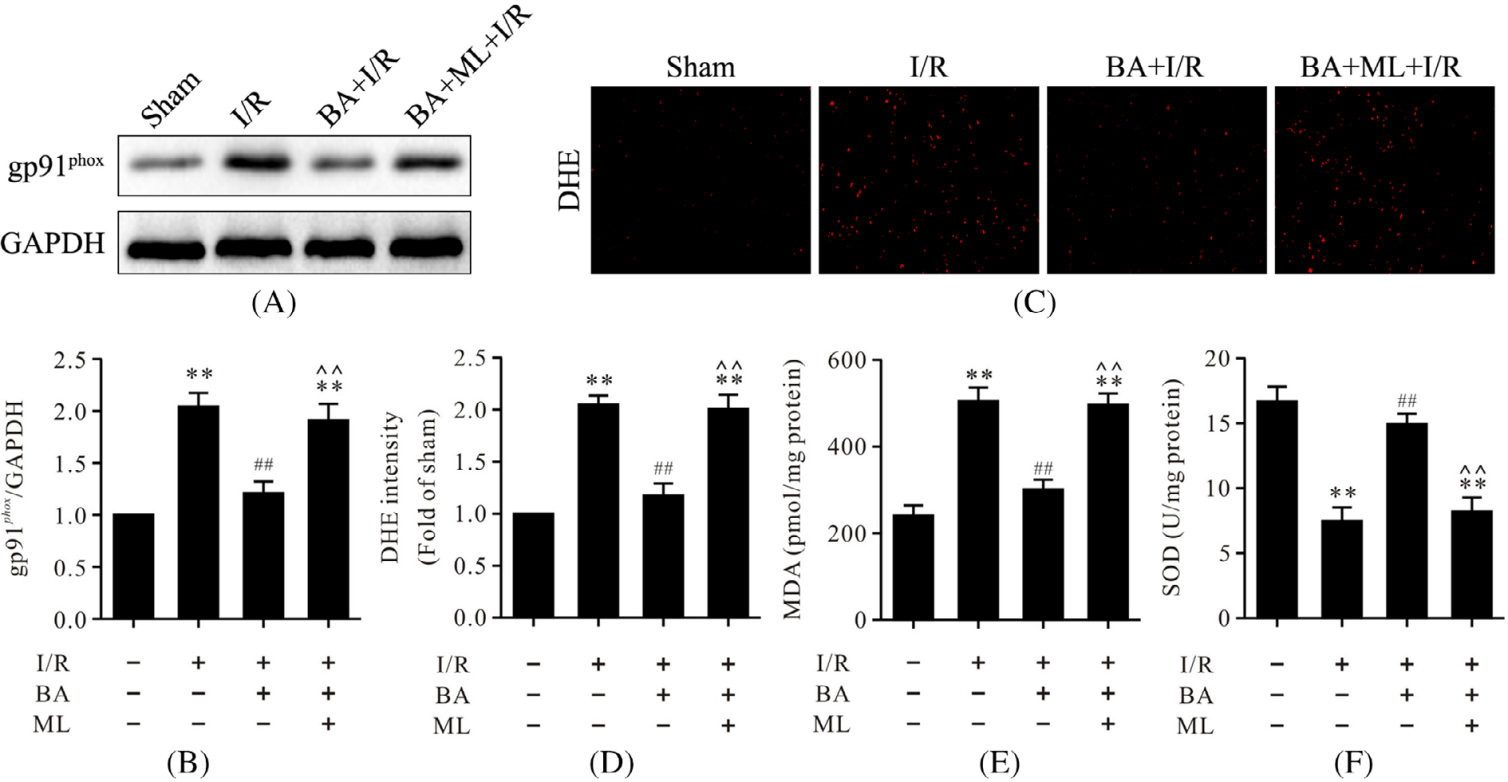
ML‐385 blunted the antioxidative effects of BA in myocardial I/R injury. (A) Representative immunoblots of gp91^phox^ and GAPDH (internal control); (B) semiquantitative analysis of gp91^phox^; (C) representative images of DHE staining of left ventricular tissue (magnification, ×400Χ); (D) DHE intensity; (E) MDA contents; and (F) SOD activity in myocardial tissues. Data are presented as the mean ± standard error of the mean, *n* = 6. **p < 0.01 versus the sham group. ^##^ *P* < 0.01 versus the I/R group; **^^** *P* < 0.01 versus the BA+I/R group. BA, berbamine; I/R, ischemia/reperfusion; DHE, dihydroethidium; GAPDH, glyceraldehyde 3‐phosphate dehydrogenase; MDA, malondialdehyde; ML, ML‐385

To further confirm whether the antiapoptotic and antioxidative effects of BA are associated with Nrf2 in myocardial I/R, ML‐385, a specific Nrf2 inhibitor, was used. As shown in Figures 5 and 6, myocardial I/R led to the abundant expressions of cleaved caspase‐3, cytosolic cytochrome c, and gp91^phox^ and increased the levels of MDA and ROS; it also led to an increase in the apoptotic index. Conversely, treatment with BA significantly reduced the expressions of cleaved caspase‐3, cytosolic cytochrome c, gp91^phox^, and the levels of MDA and ROS; it also led to a decrease in the apoptotic index. However, the results showed that ML‐385, a specific inhibitor of Nrf2, inhibited antiapoptotic and antioxidative effects of BA in myocardial I/R, suggesting that the antiapoptotic and antioxidative effects of BA are related to Nrf2 activation.

### 3.6 ML‐385 abolished berbamine‐induced alleviation of infarct size and cardiac function during myocardial ischemia/reperfusion

**FIGURE 7:**
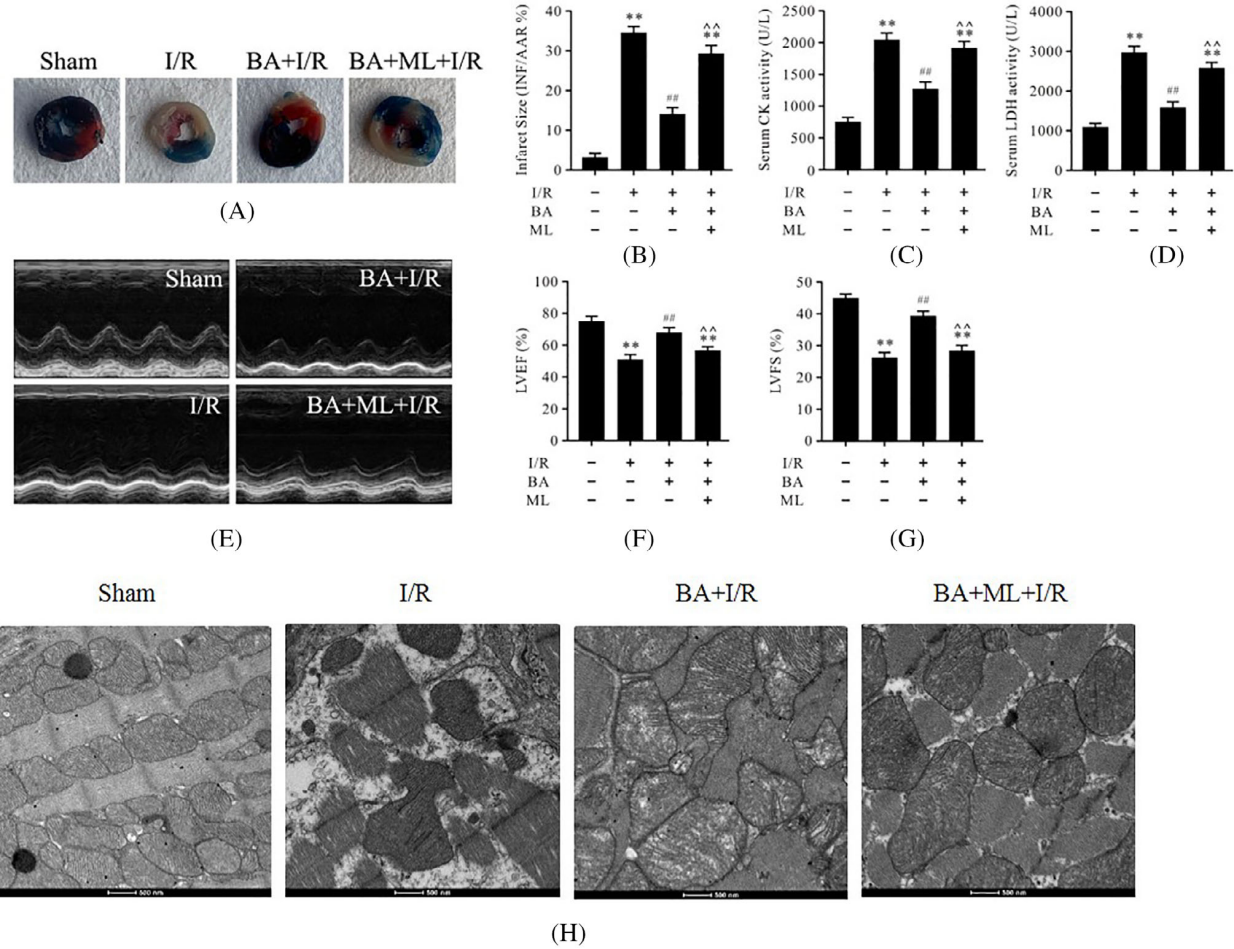
ML‐385 prevented the cardioprotective effects of BA in myocardial I/R. (A) Evans blue and TTC staining of heart slices; (B) myocardial infarct size; (C) serum CK activity; (D) serum LDH activity; (E) representative images of motion‐mode echocardiography; (F) LVEF; (G) LVFS; and (H) mitochondrial morphology and state. Data are presented as the mean ± standard error of the mean, *n* = 6. **p < 0.01 versus the sham group, ^##^ *p* < 0.01 versus the I/R group, **^^** *p* < 0.01 versus the BA+I/R group. BA, berbamine; CK, creatine kinase; LVEF, left ventricular ejection fraction; LVFS, left ventricular fraction shortening; I/R, ischemia/reperfusion; ML, ML‐385; TTC, triphenyltetrazolium chloride

To investigate whether Nrf2 inhibition could abolish the cardioprotective effects of BA in myocardial I/R, ML‐385 was used in these experiments and then cardiac function and myocardial injury were detected. As shown in Figure 7, myocardial I/R led to the enlarged infarct size and the serum CK and LDH activities (Figure 7A–D). In contrast, administration of BA reduced infarct size and decreased the serum CK and LDH activities after myocardial I/R (Figure 7A–D). Further, I/R treatment dramatically worsened cardiac function by decreasing LVEF and LVFS (Figure 7E–G). BA consistently improved cardiac function significantly, which was markedly reversed by treatment with ML‐385 (Figure 7E–G). Nevertheless, these protective effects of BA were also abolished by treatment with ML‐385, which suggested that the cardioprotective effects of BA in myocardial I/R was related to Nrf2 activity. The results of the electron microscopic examination showed that the morphology and structure of the mitochondria in the sham group were normal and that the density of the matrix was uniform and arranged neatly. In the I/R group, the mitochondria were swollen and deformed, the cristae were broken and had disappeared, and the arrangement was disordered. Compared with that of the I/R group, the morphology of the mitochondria in the BA + I/R group recovered, the mitochondrial cristae were roughly normal and slightly swollen, and the structure was improved whereas ML‐385 could reverse these effects induced by BA (Figure 7H).

### 3.7 ML‐385 attenuated berbamine‐induced increase of Nrf2 nuclear translocation but had little effect on 5′ adenosine monophosphate‐activated protein kinase activation in myocardial ischemia/reperfusion injury

**FIGURE 8:**
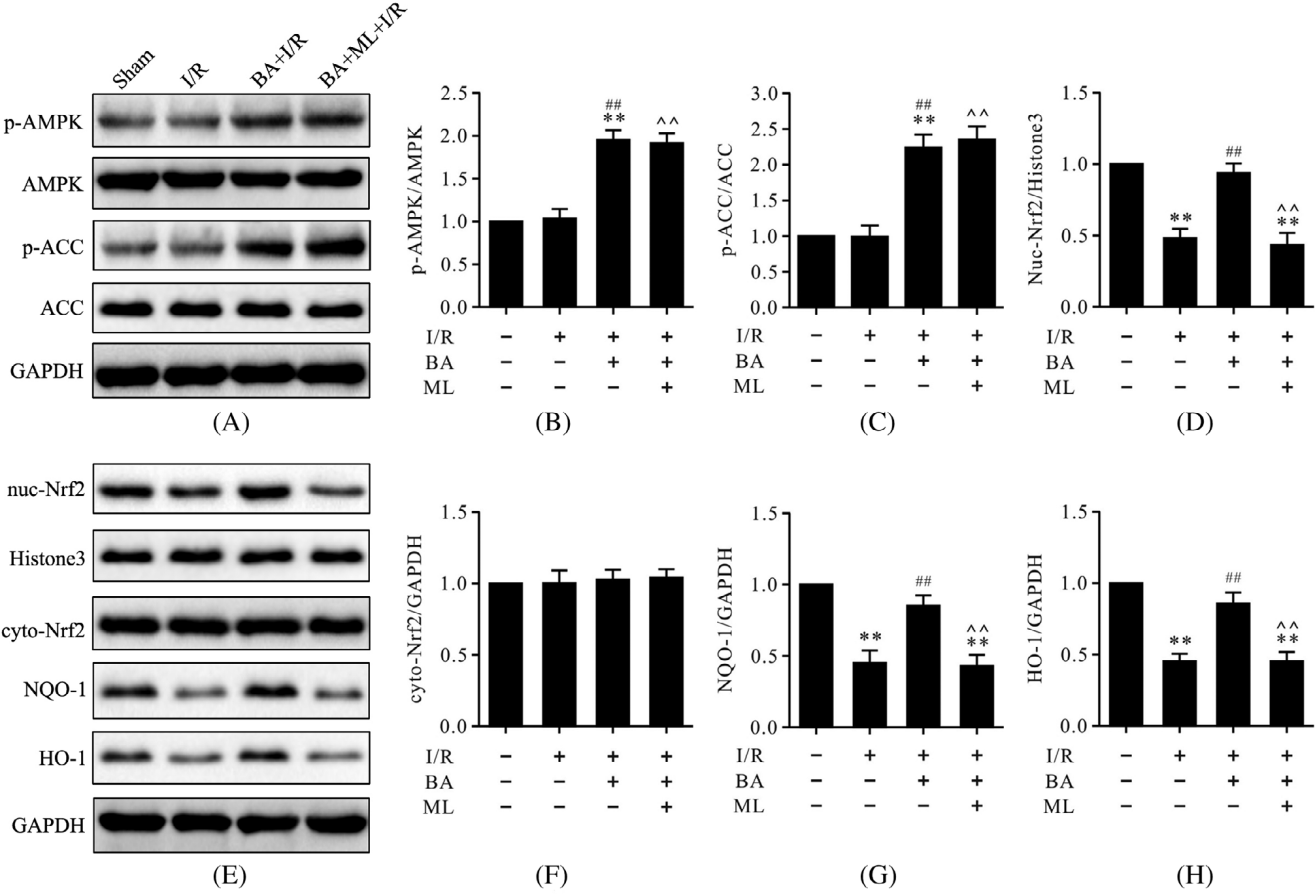
ML‐385 retarded BA‐induced Nrf2 nuclear translocation but had little effect on AMPK activation in myocardial I/R injury. (A) Representative immunoblots of p‐AMPK, AMPK, p‐ACC, ACC, and GAPDH (internal control); (B,C) semi‐quantitative analysis of p‐AMPK and p‐ACC; (D) semi‐quantitative analysis of nuc‐Nrf2; (E) representative immunoblots of nuc‐Nrf2, histone H3 (internal control), cyto‐Nrf2, NQO‐1, HO‐1, and GAPDH (internal control); (F–H) semi‐quantitative analysis of cyto‐Nrf2, NQO‐1, and HO‐1. Data are presented as the mean ± standard error of the mean, *n* = 6. **p < 0.01 versus the sham group; ^##^ *p* < 0.01 versus the I/R group; **^^** *p* < 0.01 versus the BA+I/R group. BA, berbamine; cyto‐Nrf2, cytoplasmic Nrf2; GAPDH, glyceraldehyde 3‐phosphate dehydrogenase; I/R, ischemia/reperfusion; ML, ML‐385; nuc‐Nrf2, intranuclear Nrf2

To further illustrate the correlation between AMPK and Nrf2, we performed immunoblots of the proteins p‐AMPK, p‐ACC, intranuclear Nrf2, cytoplasmic Nrf2, NQO‐1, and HO‐1. As shown in Figure 8, consistent with our previous results, western blot analysis showed that I/R treatment significantly reduced the protein levels of intranuclear Nrf2, NQO‐1, and HO‐1 but had little effect on the protein levels of p‐AMPK, p‐ACC, and cytoplasmic Nrf2 (Figure 8). Treatment with BA consistently increased the protein levels of p‐AMPK, p‐ACC, NQO‐1, HO‐1, and intranuclear Nrf2 (Figure 8). However, ML‐385 could reverse the Nrf2 nuclear translocation induced by BA but had little effect on the protein levels of p‐AMPK and p‐ACC (Figure 8). These data indicated that AMPK functioned upstream of Nrf2 signaling in BA‐mediated cardioprotective effects against myocardial I/R.

## 4 DISCUSSION

In this study, we demonstrated that BA could alleviate myocardial I/R injury by improving cardiac function and reducing apoptotic cell death. These findings are consistent with the results of previous studies. Furthermore, the cardioprotective effects of BA against myocardial I/R injury were associated with inhibiting apoptosis and oxidative stress. More importantly, compound C, the AMPK specific inhibitor, blocked AMPK activation, and Nrf2 nuclear translocation and inhibited the antiapoptotic and antioxidative effects of BA, suggesting that AMPK/Nrf2 signaling activation was essential for BA‐alleviated myocardial I/R injury. In addition, BA protected against myocardial I/R injury by activating AMPK/Nrf2 as further demonstrated by the evidence that the specific Nrf2 inhibitor ML‐385 abolished Nrf2 nuclear translocation, the antiapoptotic and antioxidative effects, and cardioprotection induced by BA but had little effect on AMPK activation. These results demonstrate that BA protects against myocardial I/R injury by inhibiting apoptosis and oxidative stress through activating the AMPK/Nrf2 pathway.

Despite timely reperfusion using primary percutaneous coronary intervention, thrombolytic therapy is the most effective treatment for patients with ST‐segment elevation myocardial infarction. Morbidity and mortality remain extremely high as evidenced by death reported in 7% and heart failure in 22% of patients at 1 year after the event.^32^ Apoptosis, known as programmed cell death, contributes to cell death during myocardial I/R injury.^33^, ^34^ Results of a previous study indicated that apoptosis is the predominant form of cardiomyocyte death, which is evident at the infarcted border zone.^35^ Consistent with the results of other previous studies, we found that BA alleviated I/R‐induced cardiac dysfunction and myocardial injury as evidenced by increased LVEF and LVFS and decreased infarct size, and serum CK and LDH activities. Meanwhile, BA reduced apoptotic cell death as evidenced by significantly decreased levels of cleaved caspase‐3, cytosolic cytochrome c, caspase‐3 activity, and the apoptotic index. However, the AMPK specific inhibitor compound C and the specific Nrf2 inhibitor ML‐385 could noticeably inhibit the cardioprotective changes induced by BA. These results indicated that AMPK and Nrf2 might be involved in the cardioprotective and antiapoptotic effects of BA in myocardial I/R injury.

Berbamine is an extract from the roots, root bark, and stem bark of *Berberis amurensis*, a traditional Chinese herbal medicine in the berberiaceae family. It can promote hematopoietic function and increase the quantity of blood cells. It is used to prevent and treat leukopenia caused by radiotherapy and chemotherapy, benzene poisoning, radioactive substances, and drugs in patients with tumors. Berbamine has a curative effect on silicosis, leukopenia, thrombocytopenia, essential hypertension, and so forth. It can also be used as a muscle relaxant for traditional Chinese medicine anesthesia.

Oxidative stress is a prominent pathological feature and plays an important role in the pathogenesis of myocardial I/R injury.^36^, ^37^ Disruption of oxidant‐antioxidant homeostasis is the main factor responsible for cellular oxidative stress. Antioxidant enzymes such as SOD and glutathione peroxidase protect cardiomyocytes from oxidative damage.^38^ Furthermore, BA is well known as an oxidation inhibitor that exerts antioxidant properties in cardiovascular, metabolic, and neurodegenerative diseases.^39^, ^40^, ^41^ In this regard, we presume that the cardioprotective effects of BA are related to the mitigation of oxidative stress and mitochondrial damage in myocardial I/R. In the current study, the results showed that the levels of gp91^phox^, ROS, and MDA significantly increased in the I/R group whereas SOD activity decreased compared to the sham group. At the same time, mitochondrial morphological and functional damage was found in the I/R group compared to the sham group. As expected, I/R treatment with BA decreased the levels of gp91^phox^, ROS, and MDA and improved SOD activity. Nevertheless, compound C and ML‐385 could obstruct BA‐induced antioxidative effects in myocardial I/R injury, suggesting that the antioxidative effects of BA and of mitochondrial morphological and functional damage were related to AMPK and Nrf2.

AMPK, a master energy‐sensing serine/threonine kinase, functions as a cardiac energy sensor and maintains energy homeostasis in physiological and pathological conditions.^16^ AMPK is activated in response to cellular metabolic stresses, and then phosphorylates and inactivates ACC, thereby permitting carnitine palmitoyltransferase 1 activity and thus promoting the oxidation of fatty acids.^42^ Furthermore, there has been an increasing awareness that AMPK responds to circulating hormones and local autocrine and paracrine factors.^43^, ^44^ In this study, we employed a myocardial I/R model to confirm that treatment with BA could regulate AMPK and Nrf2. Our results demonstrated that treating I/R with BA significantly increased phosphorylation of AMPK and ACC and increased the levels of intranuclear Nrf2, NQO‐1, and HO‐1. However, compound C could obstruct the BA‐dependent regulation of AMPK and Nrf2 in myocardial I/R, validating the concept that BA could regulate the AMPK/Nrf2 signaling pathway in myocardial I/R injury. Intriguingly, ML‐385 could also thwart BA‐dependent regulation of Nrf2 but had little effect on AMPK activation, suggesting that Nrf2 serves as a downstream activator of AMPK signaling in BA‐induced cardioprotective effects against myocardial I/R injury.

Nrf2, a ubiquitously expressed transcription factor, regulates the transcription of a range of target genes in response to oxidative stress in basal and pathological conditions.^45^ It has previously been shown that Nrf2 plays a crucial role in the cardiovascular system by regulating antioxidant enzymes such as NQO‐1 and HO‐1.^46^ Accumulating evidence has shown that Nrf2 activation by some interventions can lessen myocardial infarct size and cardiac hypertrophy in rodents exposed to I/R and pressure overload, respectively.^47^, ^48^ We found that the levels of intranuclear Nrf2, NQO‐1, and HO‐1 were significantly decreased in the I/R group. More importantly, treatment with BA dramatically increased the levels of intranuclear Nrf2, NQO‐1, and HO‐1. Predictably, both compound C and ML‐385 could inhibit the levels of intranuclear Nrf2, NQO‐1, and HO‐1 induced by BA. These results further confirmed that the AMPK/Nrf2 signaling pathway is involved in the cardioprotective effects of BA in myocardial I/R injury.

## 5 CONCLUSIONS

Our study results have demonstrated that BA alleviates myocardial I/R injury by improving mitochondrial morphology and by reducing apoptosis and oxidative stress. More importantly, our results also indicate that the AMPK/Nrf2 signaling pathway plays a pivotal role in mediating the cardioprotective effects of BA in myocardial I/R injury and improving mitochondrial morphology and state. Therefore, our results suggest that BA is a promising therapeutic intervention for patients with ischemic heart disease.

## CONFLICT OF INTERESTS

The authors have no conflict of interests to declare.

## AUTHOR CONTRIBUTIONS

Chennian Xu and Yang Liu designed the research. Chennian Xu, Yang Liu, and Ping Jin conducted the experiments. Mengen Zhai, Zhenge Fan, and Yang Liu analyzed and interpreted the data. Chennian Xu and Jian Yang wrote the manuscript. The study was supervised by Jian Yang and Lifang Yang. All authors read and approved the final manuscript.

## ETHICS STATEMENT

The experimental procedures involving animals were performed in accordance with the guidelines of the Xijing ethics committee and local laws and policies. All of the procedures were approved by the Food and Veterinary Service, China.

## Data Availability

The data used in this study are available from the corresponding author upon request.
